# A self-knowledge distillation-driven CNN-LSTM model for predicting disease outcomes using longitudinal microbiome data

**DOI:** 10.1093/bioadv/vbad059

**Published:** 2023-05-18

**Authors:** Daryl L X Fung, Xu Li, Carson K Leung, Pingzhao Hu

**Affiliations:** Department of Computer Science, University of Manitoba, Winnipeg, MB R3T 2N2, Canada; Division of Biostatistics, Dalla Lana School of Public Health, University of Toronto, Toronto, ON M5T 3M7, Canada; Department of Computer Science, University of Manitoba, Winnipeg, MB R3T 2N2, Canada; Department of Computer Science, University of Manitoba, Winnipeg, MB R3T 2N2, Canada; Division of Biostatistics, Dalla Lana School of Public Health, University of Toronto, Toronto, ON M5T 3M7, Canada; Department of Biochemistry and Medical Genetics, University of Manitoba, Winnipeg, MB R3E 0J9, Canada; Department of Biochemistry, Western University, London, ON N6A 5C1, Canada

## Abstract

**Motivation:**

Human microbiome is complex and highly dynamic in nature. Dynamic patterns of the microbiome can capture more information than single point inference as it contains the temporal changes information. However, dynamic information of the human microbiome can be hard to be captured due to the complexity of obtaining the longitudinal data with a large volume of missing data that in conjunction with heterogeneity may provide a challenge for the data analysis.

**Results:**

We propose using an efficient hybrid deep learning architecture convolutional neural network—long short-term memory, which combines with self-knowledge distillation to create highly accurate models to analyze the longitudinal microbiome profiles to predict disease outcomes. Using our proposed models, we analyzed the datasets from Predicting Response to Standardized Pediatric Colitis Therapy (PROTECT) study and DIABIMMUNE study. We showed the significant improvement in the area under the receiver operating characteristic curve scores, achieving 0.889 and 0.798 on PROTECT study and DIABIMMUNE study, respectively, compared with state-of-the-art temporal deep learning models. Our findings provide an effective artificial intelligence-based tool to predict disease outcomes using longitudinal microbiome profiles from collected patients.

**Availability and implementation:**

The data and source code can be accessed at https://github.com/darylfung96/UC-disease-TL.

## 1 Introduction

Our human body harbors highly dynamic and distinct microbial communities called the human microbiome (Handelsman, 2004). The microbiome changes over time due to diet, antibiotics and age. The microbiome can be influenced by infections or medical interventions over time resulting in a different microbial composition (Gilbert *et al.*, 2018). The recent advancements in technology have enabled cheaper sequencing that increased the amount of microbiome sequencing data publicly available. This has enabled the availability of large amount of microbiome sequencing data paired with disease phenotypes from patients with different complex diseases (Wang *et al.*, 2012). Hence, researchers have been motivated to use machine learning models and deep learning models to extract the features from the human microbiome data to predict their disease outcomes and phenotypes.

LaPierre *et al.* (2019) reviewed a variety of machine learning models and feature extraction methods used to analyze Type 2 Diabetes microbiome data. In microbiome-based disease prediction, the most commonly used machine learning models are support vector machine (SVM) (Cortes and Vapnik, 1995) and Random Forest (RF) (Breiman, 2001). They are able to output the most informative features that include the microbes or the functional elements, which contribute the most to the disease prediction. The informative features can be used to provide insight into the relationship between the microbiome and the disease. In addition, deep learning has also been used to predict disease outcomes through microbiome sequencing data. Deep learning can learn more complex relationships between the input and the output. However, the interpretability of deep learning models can be reduced as more complex functions are applied.

Rahman and Rangwala (2018) proposed to use a new Multi-Instance Learning (MIL) to determine the clinical phenotype from microbiome sequencing data. They tested their model using the data from a liver cirrhosis study (Qin *et al.*, 2010) and an inflammatory bowel disease (IBD) study (Qin *et al.*, 2014). They showed that their model was able to outperform other comparative MIL models (Andrews *et al.*, 2003; Bunescu and Mooney, 2007; Kotzias *et al.*, 2015) and non-MIL methods (Pasolli *et al.*, 2016; Qin *et al.*, 2014). MIL is a weakly supervised approach that contains bags of instances with labels. In a MIL approach, rather than having each instance containing a label, there will be a bag of instances where the bag will contain the label. In a binary case, bags that contain at least one positive instance will be assigned with a label of 1. Bags that contain all negative instances will be assigned a label of 0. Using their new approach, they were able to achieve an area under the receiver operating characteristic curve (ROC-AUC) score of 0.8442 for IBD dataset and the ROC-AUC score of 0.9272 for the liver cirrhosis dataset. ROC-AUC is a model performance measure with 1 representing a perfect model performance. However, the methods showed only to extract microbial features from a single time-point, missing valuable information of the temporal changes of the microbiome. As the human microbiome is highly dynamic in nature, the temporal characteristics of the microbial feature may contain richer information for disease prediction.

Sharma and Xu (2021) proposed a novel deep learning framework called ‘phyLoLSTM’ that uses convolutional neural network (CNN) to extract the microbial features and long short-term memory (LSTM) to analyze the temporal dependency in microbiome sequencing data for predicting disease outcomes. They also proposed a novel data pre-processing method that is able to handle the variable time points in each subject, and weight balancing to handle the imbalanced disease classes in the datasets. They tested their model on simulated dataset and real datasets, including DIABIMMUNE (Vatanen *et al.*, 2016) and DiGiulio (DiGiulio *et al.*, 2015). The DIABIMMUNE study includes three country cohorts that determined food allergy outcomes of subjects to milks, peanuts, eggs and overall. DiGiulio study contains the preterm delivery as outcomes based on the microbial taxa from vagina, saliva, distal gut and gum. They showed that their method was able to achieve 0.897 ROC-AUC on simulated dataset, 0.713 ROC-AUC and 0.762 ROC-AUC on the DIABIMMUNE and the DiGiulio datasets, respectively.

Chen *et al.* proposed a deep learning network, specifically Gated Recurrent Unit (GRU) (Cho *et al.*, 2014), that uses longitudinal microbiome data to predict the human host status (Chen *et al.*, 2021). They tested their method on both semi-synthetic and real datasets (Brooks *et al.*, 2017; David *et al.*, 2014; Hall *et al.*, 2017; Heintz-Buschart *et al.*, 2016; Pasolli *et al.*, 2017; Raymond *et al.*, 2016; Shao *et al.*, 2019; Vatanen *et al.*, 2016; Vincent *et al.*, 2016). The semi-synthetic dataset is from Bokulich *et al.* 92016) and the same setting and parameters based on a Microbiome Interpretable Temporal Rule Engine (MITRE) (Bogart *et al.*, 2019) were used. A pipeline of data pre-processing was undergone to further improve the performance of the deep learning networks. They were able to achieve high performance compared with other baseline classifiers and improve the evaluation time taken to classify the subjects.

Metwally *et al.* (2019) investigated using LSTM to predict food allergies using the microbial features from longitudinal human microbiome profiles. They compared their model against machine learning models, such as Hidden Markov Model (Rabiner and Juang, 1986), Multi-Layer Perceptron Neural Network (Tadeusiewicz, 1995), Support Vector Machine (Cortes and Vapnik, 1995), least absolute shrinkage and selection operator (LASSO) Regression (Tibshirani, 1996) and Random Forest (Ho, 1995). They also reduced the microbial features by using sparse autoencoder (Ng, 2011) to extract the latent representations of the microbial features in addition to using Minimum Redundancy Maximum Relevance (mRMR) and ranking based on variance for training of the LSTM network. The model based on the latent representations of sparse autoencoder achieved the highest performance with a ROC-AUC of 0.67 compared with those using the microbial features selected using mRMR and ranking based on variance. The study showed that the learning of LSTM can be useful to capture the temporal information in the microbiome data for improving the prediction of the subjects’ clinical outcomes.

García-Jiménez *et al.* (2021) integrated deep learning techniques to condense the microbial composition into a deep latent space representation with less but rich latent features from microbiome data. They showed that using deep learning techniques, they are able to predict the microbiome composition using environmental factors including temperature, plant age and precipitation. They showed that transfer learning was able to improve the performance further. They experimented with different orders to transfer the features from Maarastawi dataset (Maarastawi *et al.*, 2018) to the Walters *et al* dataset (Walters *et al.*, 2018). Using Pearson correlation and Bray–Curtis dissimilarity, which measure the similarity or dissimilarity between the observed microbiome data and the predicted microbiome data, and the best transfer features is the phylum order achieving 0.9451 Pearson correlation and 0.1833 Bray–Curtis dissimilarity.

As deep learning networks require a huge amount of data to prevent from overfitting, deep learning networks are more prone to overfitting when trained on the microbiome datasets due to its small size nature. Zhang *et al.* (2019) proposed a general training framework called self-distillation, which improves the model performance of deep learning networks. Self-distillation is similar to knowledge distillation where a student network’s distribution is encouraged to share similar distributions or weight features with the teacher network’s weight features. However, instead of learning from a teacher network, self-distillation learns from its network itself. The network is separated into individual shallow sections, and the learned knowledge of the deeper parts of the network is shared with the shallow sections of the network. They showed that this technique was able to improve the model prediction accuracy at an average of 2.65%, having a variation between 0.61% and 4.07% using ResNeXt (Xie *et al.*, 2017) and VGG19 (Simonyan and Zisserman, 2015), respectively.

Kim *et al.* (2020) proposed a simple but effective regularization method by using a self-distillation called progressive self-knowledge distillation (PS-KD). It does self-distillation by progressively regularizing the training using the model’s previous epoch weights. The model’s previous epoch weights act as the teacher model to distill the knowledge into the student model (itself). PS-KD is applicable to any kinds of supervised learning approaches with hard targets, and can be used to further generalize the performance through combining with existing regularization methods. Their evaluation showed that PS-KD achieved high accuracy and high-quality confidence estimation as measured through ordinal ranking and calibration. They experimented their method on multiple tasks including object detection, image classification and machine translation, and showed that their method was able to improve the performance of models in all three tasks.

The major challenge with the longitudinal human microbiome data is that there exists missing information along the different time points for different subjects, causing uneven distribution of the microbiome data. To mitigate the problem of missing information, we will use padding in sequence, where the missing information is padded. To address the large number of operational taxonomic units (OTUs) in the microbiome profiles but the small number of subjects collected at each time point, we develop an efficient hybrid deep learning architecture convolutional neural network—long short-term memory (CNN-LSTM). It is combined with self-knowledge distillation to create high accurate models to analyze the longitudinal human microbiome data to predict disease outcomes, where the CNN is used to extract informative microbial features and the LSTM is used to understand the temporal dynamic of the human microbiome.

## 2 Methods

### 2.1 Datasets and data preprocessing

#### 2.1.1 Longitudinal microbiome data

The datasets that we use to evaluate our models are PROTECT (Predicting Response to Standardized Pediatric Colitis Therapy) study (Hyams *et al.*, 2017) and DIABIMMUNE dataset (Vatanen *et al.*, 2016).

PROTECT study consists of 428 subjects with new-onset pediatric ulcerative colitis disease from USA and Canada, and was monitored over the course of a year. Patients did not receive any treatments at week 0, and were assigned into one of two treatments—either 5-aminosalicylic acid (mesalamine) or oral/intravenous (IV) corticosteroid (CS) followed by 5-aminosalicylic acid (mesalamine). The disease progression was monitored with the treatment progression. Within the 428 subjects, there are 405 subjects where stool and rectal samples were collected for microbiome sequencing. The samples were collected on week 0, week 4, week 12 and week 52. Clinical and metadata were collected throughout the year, including gender, ethnicity, age, PUCAI, treatment, disease progression, stool consistency, fecal calprotectin and the extent of the disease involvement. Patients are between the age of 4 and 17 with 48% of them being females and 52% being males. The stool samples were sequenced using 16s rRNA gene amplicon sequencing technology on Illumina Miseq Platform (Schirmer *et al.*, 2018). The dataset includes 1015 OTUs generated using the 16S bioBakery workflow built with AnADAMA2 (McIver *et al.*, 2018).

DIABIMMUNE is a study that aims to explore the role of hygiene in the development of Type 1 Diabetes and other autoimmune diseases. The dataset contains families from three different countries: Finland, Estonia and Russia. Each subject is an early infant before the first 6 months of age and they were followed until the age of 3. There are 74 infants as subjects in each country. Three years of monthly stool samples, laboratory assays and questionnaires that include breastfeeding, diet, allergies, family history, infections, clinical examinations and use of drugs were collected. A total of 1584 stool samples were sequenced using 16s rRNA gene amplicon sequencing technology on Illumina HiSeq 2500 Platform. The dataset includes 282 OTUs generated using QIME v1.8.0.

For both datasets, we downloaded the processed OTUs level data from the original studies, which has been normalized as relative abundance.

#### 2.1.2 Data pre-processing

As the microbiome sequencing data contain invariant timepoints caused by missing information from the subjects at some time points, and we will use LSTM to model the longitudinal microbiome data (see below Section 2.2.1), it is useful to use padding techniques for imputing the missing data. The forward feed and back propagation operations in LSTM will improve the missing value estimations over the padded initial values. Hence, we propose to use several methods to solve the issue.

The first method that we used is to pad the data in sequence. For instance, in the case of the PROTECT study, if a subject contains week 0 and week 52 but misses week 4 and week 12, the subject will be padded with 0s on week 4 and week 12, maintaining the sequence and the order of the longitudinal data of the subject. The second method that we used is to pad and mask the padded redundant data from the subject. An example of the padding techniques is shown in Table 1. In addition, we also passed the microbiome sequencing data into a dimension reduction algorithm. Here we used principal component analysis (PCA) to determine if there is an improvement on performance through selecting the top principal components of the microbiome data. We set the number of principal components to be 300.

**Table 1.** An example of our padding techniques

Pad in sequence					
	Ruminococcaceae	Peptostreptococcaceae	Alcaligenaceae	…	Porphyromonadaceae
Week 0	0. 29747	0.00381	0.00114	…	0.18839
Week 4	0	0	0	0	0
Week 12	0	0	0	0	0
Week 52	0.00168	0.00839	0. 00839	…	0.50269

Pad and mask					
	Ruminococcaceae	Peptostreptococcaceae	Alcaligenaceae	…	Porphyromonadaceae
Week 0	0. 29747	0.00381	0.00114	…	0.18839
Week 52	0.00168	0.00839	0. 00839	…	0.50269
0	0	0	0	0	0
0	0	0	0	0	0

*Note*: Both padding techniques are done on the same subject that contains missing data in week 4 and week 12.

### 2.2 Methods

#### 2.2.1 Self-knowledge distillation modelling framework

##### 2.2.1.1 Long-short term memory model

The LSTM network learns temporal information of the data. It consists of three different gates: input gate, output gate and the forget gate. The gates control how much of the information needed to be passed into the next time step. Sigmoid is used as a gating mechanism to control the input to be between the value of 0 and 1. The forget gate is defined as follows: where $W_{f}$ is the weights for the forget gate to transform the input at the current time step,$\;x_{t}$ is the input at the current time step, $U_{f}$ is the weights for the forget gate to transform the hidden state from the previous time step, $h_{t-1}$ is the hidden state from the previous time step, $b_{f}$ is the bias and σ is a sigmoid function that squashes the input to value between 0 and 1.


(1) $$f_{t}=\sigma (W_{f}x_{t}+U_{f}h_{t-1}+b_{f}),$$

The input gate is defined as follows: where $W_{i}$ is the weights for the input gate to transform the input at the current time step, $x_{t}$ is the input at the current time step, $U_{i}$ is the weights for the input gate to transform the hidden state from the previous time step, $h_{t-1}$ is the hidden state from the previous time step, $b_{i}$ is the bias and σ is a sigmoid function.


(2) $$i_{t}=\sigma (W_{i}x_{t}+U_{i}h_{t-1}+b_{i}),$$

The output gate is defined as follows: where $W_{o}$ is the weights for the output gate to transform the output at the current time step, $x_{t}$ is the input at the current time step, $U_{o}$ is the weights for the output gate to transform the hidden state from the previous time step, $h_{t-1}$ is the hidden state from the previous time step, $b_{o}$ is the bias and σ is a sigmoid function.


(3) $$o_{t}=\sigma (W_{o}x_{t}+U_{o}h_{t-1}+b_{o}),$$

The cell state at the current time step is calculated as: where $c_{t-1}$ is the cell state from the previous time step. If $c_{t}$ is at time t=0, then $c_{t-1}$ is 0. $i_{t}$ is the output of the input gate at the current time step, $W_{c}$ is the weight for the cell state, $x_{t}$ is the input at the current time step, $U_{c}$ is the weight for the cell state to transform the hidden state from the previous time step, $h_{t-1}$ is the hidden state from the previous time step, $b_{c}$ is the bias and tanh is a Tan*h* function that squashes values between –1 and 1. The cell state helps to control how much to forget from the previous time step and how much to include the current time step information into the cell state.


(4) $$c_{t}=f_{t}*c_{t-1}+i_{t}*\tan h\left(W_{c}x_{t}+U_{c}h_{t-1}+b_{c}\right),$$

The hidden state of the current time step is then calculated as:


(5) $$h_{t}=o_{t}*\sigma (c_{t})$$

The last output of the LSTM network is then flattened into a vector and fed into a multi-layer perceptron with a sigmoid activation to classify the disease task.


(6) $$y=\mathrm{sigmoid}({W_{y}h}_{t}+b_{y}).$$

##### 2.2.1.2 CNN-LSTM model

The CNN-LSTM model uses a combination of CNN and LSTM to classify the disease task. The model first uses CNN to extract the features of the gut microbiome data at each time point and pass the extracted features as input to the LSTM network. The CNN uses a kernel to extract features based on a group of input features. The equation for the extraction of the input using CNN is: where $w_{i,j}^{l}$ is the weights in layer *l* at row i and column *j* of the kernel, $i_{d+i,e+j}^{l-1}$ is the output from the previous layer at row *d + i* and column *e + j* and $b_{d,e}^{l}$ is the bias in layer *l* for the output at row *d* and column *e*.


(7) $$O_{d,e}=\sum_{i}^{k}\sum_{j}^{k}w_{i,j}^{l}*i_{d+i,e+j}^{l-1}+b_{d,e}^{l},$$

CNN-LSTM contains two pipelines. First, the gut microbiome data are fed into the CNN (Krizhevsky* et al.*, 2012) to extract features. The extracted features from the CNN are then fed into the LSTM (Hochreiter and Schmidhuber, 1997) to learn the temporal dynamics of the gut microbiome. We also evaluated with and without a dimension reduction algorithm, PCA (Pearson, 1901), to determine if PCA could improve the performance of the deep learning models. We used the first 300 principal components to feed into the deep learning models and compared the results against each other.

##### 2.2.1.3 Self-distillation model

We implemented self-distillation on the LSTM and CNN-LSTM models. We implemented multiple shallow classifiers and branched them out from the layers before the main classifier in the LSTM network. The multiple shallow classifiers classify the disease task just like the main classifier. We integrated additional loss functions to the shallow classifier to improve the features learned by the network: (i) Cross entropy loss function is used to improve the shallow classifier predictions to the ground truth labels. (ii) KL divergence is used to minimize the distribution between the prediction of the shallow classifier and the main classifier. (iii) L2 loss is incurred on the shallow classifier layer right before the output layer with the layer right before the output layer of the main classifier.

The cross-entropy loss function is defined as:


(8) $$\mathrm{Cross}\;\mathrm{entropy}\;\mathrm{loss}=-\frac{1}{m}\sum_{i=1}^{m}y_{i}\;*\;\log({\hat{y}}_{i}).$$

The KL divergence loss is: where $y_{i}$ is the ground truth label, ${\hat{y}}_{i}$ is the prediction and *m* is the total number of samples. The distribution between the prediction of the shallow classifier and the main classifier is minimized to make the shallow classifiers’ distributions as similar as possible to the main classifier’s distribution.


(9) $$\mathrm{KL}\;\mathrm{Divergence}\;\mathrm{loss}=-\sum_{i=1}^{m}y_{i}\;*\;\mathrm{loglog}\;\left(\frac{y_{i}}{{\hat{y}}_{i}}\right),$$

**Fig. 1.**
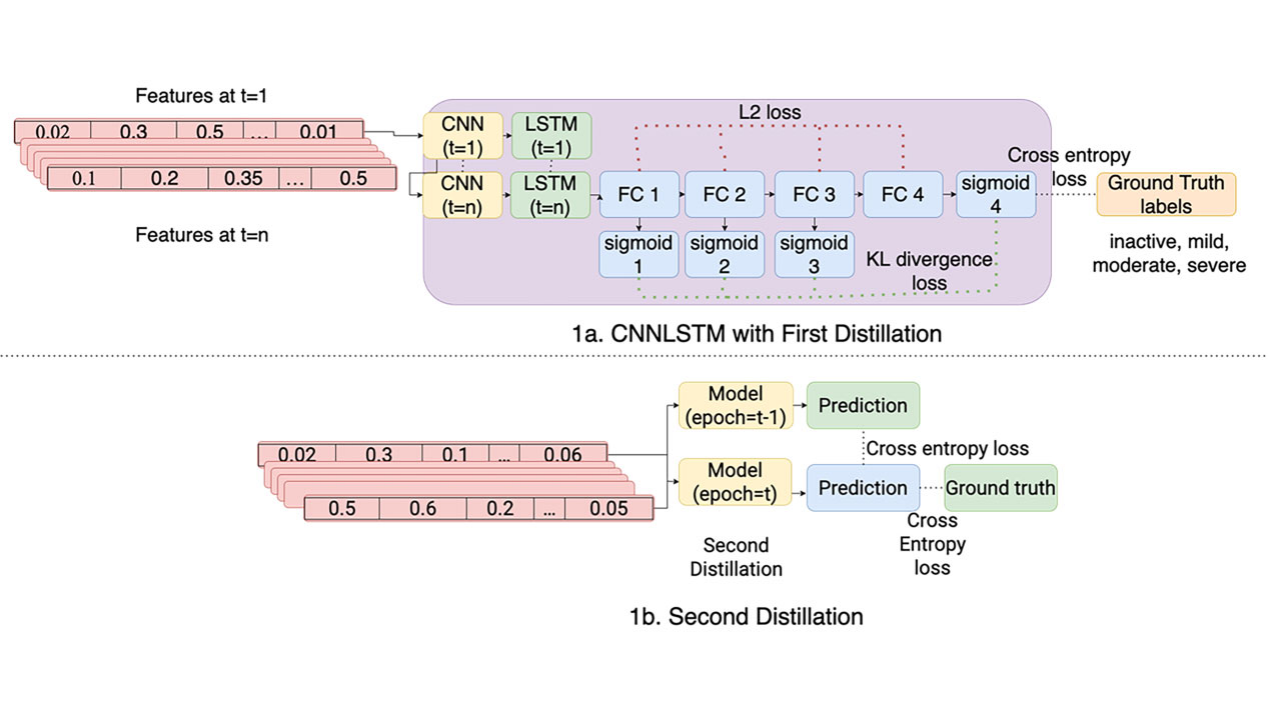
Architecture of CNN-LSTM. (**a**) The first self-distillation method consists of several sub-classifier to predict the labels of the ground truth and the prediction of the main classifier; (**b**) the second self-distillation method uses the model’s previous epoch prediction as a regularization

The L2 loss function is defined as follows: where $W_{i}^{m}$ is the weight of the layer before the main (*M*) classifier and $W_{i}^{s}$ is the weight of the layer before the shallow (*S*) classifier. Figure 1a shows the architecture of the first self-distillation method on a CNNL-STM network.


(10) $$L2\;\mathrm{loss}=\frac{1}{m}\sum_{i=1}^{m}{\left(W_{i}^{M}-W_{i}^{S}\right)}^{2},$$

Moreover, we implemented progressive self-distillation that uses its past prediction as a teacher model to have more informative training. The total loss function will become: ${{\hat{y}}_{i}}^{t}$ is the current prediction and ${{\hat{y}}_{i}}^{t-1}$ is the prediction using the weights of the model at the previous step. The α parameter determines how much of an emphasis we want to get from the loss of using the past prediction against the loss with the hard targets. As the teacher model does not have a reliable knowledge in the beginning, we start out with a low value of α and gradually increase α. We set α at the *t*-epoch as follows:


(11) $$\mathrm{Loss}=(1-\alpha )\;*\;\mathrm{CrossEntropy}\left(y,{{\hat{y}}_{i}}^{t}\right)+\alpha \;*\;\mathrm{CrossEntropy}({{\hat{y}}_{i}}^{t-1},{{\hat{y}}_{i}}^{t}),$$

(12) $$\alpha_{t}=\alpha_{T}\times \frac{t}{T\;},$$

*T* is the total epoch to train the model. Figure 1b shows the architecture of the second self-distillation approach.

#### 2.2.2 Transfer learning

Since both of the datasets were generated from patients with disorders of immune system and both of them are longitudinal microbiome data, we will undergo transfer learning from the dataset with better performance to the one with worse performance. We will run the learning of the features of the dataset with better performance first and then use the learned weights to transfer to learn the dataset with worse performance. In order to undergo transfer learning, we need to have the same features in both datasets. We first categorize the bacteria by the taxonomy class. We found that the overlaps between each taxonomy class between PROTECT study and DIABIMMUNE is as follows: kingdom: 2/2, phylum: 10/12, class: 17/24, order: 27/48, family: 41/85 and genus: 71/153. The denominator is the total number of different groups in a taxonomy class. The nominator is the total amount of overlap of the different groups in the taxonomy class between the PROTECT study and the DIABIMMUNE study. For phylum: 10/12, this shows that there are 10 groups that overlap out of 12 groups in the phylum order. Only the kingdom order has all the overlap of the taxonomy order. However, the kingdom order only contains two features which can be insufficient to learn rich information. Instead, we use the order of phylum. We only keep the overlapping features between the two datasets and remove the features that do not overlap. The overlapping features are *Synergistetes*, *Bacteroidetes*, *Verrucomicrobia*, *Actinobacteria*, *Fusobacteria*, *Tenericutes*, *Proteobacteria*, *Euryarchaeota*, *Cyanobacteria*, *Firmicutes.* The non-overlapping features are *TM7*, *Lentisphaerae.*

We removed the features that are non-overlapping and kept the overlapping features to train on PROTECT study. Once the network is trained on the PROTECT study, we transfer the weights of the network to train on the DIABIMMUNE dataset. During the transfer of the learned weights, we used several methods to fine-tune the weights on the DIABIMMUNE dataset—discriminative fine-tuning, gradual unfreezing and concat pooling. Discriminative fine-tuning uses a different learning rate for different layers in the network where the learning rate is higher in the last layer and gradually gets lesser toward the first layer. Gradual unfreezing froze all layers except the last layer and adjusted the weights of the last layer only on the first epoch. On the second epoch, the second last layer is unfrozen together with the last layer and adjusted during training. This goes on for the following epochs for all other layers. Concat pooling concatenates the mean of all the hidden states, the max of all the hidden states and the last hidden state and feeds them into the next feed forward network.

### 2.3 Model training

During training, we split the data into 10-fold cross-validation and averaged the validation result between all the folds. We used a batch size of 64 and had a total epoch of 100. We ran the experiment with LSTM and CNNLSTM along with self-distillation and transfer learning.

### 2.4 Comparison with baseline models

We also compared our model against an existing deep learning network that focuses on longitudinal gut microbiome (Metwally *et al.*, 2019) and a recurrent neural network (RNN) (Rumelhart *et al.*, 2013) by replacing LSTM to RNN in our model. Since microbial profiles contain a lot of features, they decided to extract meaningful features before feeding the features into an LSTM network. Their method consists of two modules: the first module is a feature extraction module that extracts the features from the input into a compressed latent representation, and the second module is an LSTM network that receives the compressed latent representation at each time steps to learn the temporal dynamics of the gut microbiome.

They used an autoencoder for their feature extraction module. Autoencoder is a neural network that carries out unsupervised learning by reconstructing the input data. It is very similar to a deep neural network. The main difference is that the middle layer of the autoencoder is much smaller than the rest of the network and is used to represent the latent space. An autoencoder hidden layer has the following equation: where $x_{l}$ is the *l*th hidden layer, $W_{l}$ is the weights for the previous hidden state’s layer, $x_{l-1}$ is the previous hidden layer. When l is 1, $x_{l-1}=x_{0}=X$, is the input. The last layer of the autoencoder is the reconstructed input $\hat{X}$.$\;b_{l}$ is the bias term at the current hidden layer. To prevent overfitting, they added an L2 regularization on the weights. They also enforced sparsity in the autoencoder by incorporating Kullback–Leibler (KL) divergence (Kullback and Leibler, 1951) to force only a small fraction of the neurons to be activated. The total loss function for the autoencoder is:


(13) $$x_{l}=\mathrm{ReLU}(W_{l}x_{l-1}+b_{l}),$$

(14) $$\mathrm{Autoencoder}\;\mathrm{loss}=\frac{1}{m}\sum_{i=1}^{m}{\left|\left|\;X_{i}-{\hat{X}}_{i}\right|\right|}^{2}+\lambda \sum_{l=1}^{L}|\left|W_{l}\right|{|}^{2}+\beta \sum_{n=1}^{N}KL(p|\left|{p^{\prime }}_{n}\right).$$

The first term is the reconstruction loss, $X_{i}$ is the input, and ${\hat{X}}_{i}$ is the reconstructed input. The second term is the L2 regularization term. λ is the parameter that emphasize on how much to regularize, $W_{l}$ is the weights at the l layer and *L* is the number of layers. The third term is the KL divergence where β is the parameter to emphasize on the KL divergence loss, *N* is the number of neurons in the compressed latent representation, *p* is the sparsity parameter, ${p^{\prime }}_{n}$ is the average activation of *n*th neuron in the compress latent representation. Having a lower sparsity parameter will cause the network to be more sparse.

As for the RNN network, we replaced LSTM with RNN in our model to compare as one of the baseline model. Instead of having three gates like the LSTM, RNN has a simpler equation to learn the temporal dynamics: where $W_{x}$ is the weights of the input, $X_{t}$ is the input at the current time step, $W_{h}$ is the weights of the previous hidden state, $h_{t-1}$ is the previous hidden state, $b_{l}$ is the bias term for the current time step and $a_{t}$ is the pre-activation of the current hidden state. $a_{t}$ is then fed into a tanh to convert into values between [–1, 1] before being an input to the hidden state in the next time step.


(15) $$a_{t}=W_{x}X_{t}+W_{h}h_{t-1}+b_{l}$$

(16) $$h_{t}=\tan h(a_{t}),$$

### 2.5 Model performance evaluation

After training the models, it is important to evaluate the performance of the models with each other. We measured the models’ performance by using ROC-AUC and F1 score. AUC is calculated through using a ROC curve which measures the performance of models with different thresholds. The AUC is obtained by calculating the area under the ROC curve. The higher the AUC, the better the model’s performance is. The F1 score can be calculated as follows:


(17) $$F1\;\mathrm{score}=2\;\times \;\frac{\mathrm{precision}\times \mathrm{recall}}{\mathrm{precision}+\mathrm{recall}}=\;\frac{\mathrm{tp}}{\mathrm{tp}+\;\frac{1}{2}\left(\mathrm{fp}+\mathrm{fn}\right)}.$$

Here, *tp* refers to true positive, *fp* refers to false positive, *fn* refers to false negative. True positive are labels that are correctly classified by the model, false positive are positive labels that are falsely classified by the model, and false negative are negative labels that are falsely classified by the model.

## 3 Results

### 3.1 Model training results

**Fig. 2.**
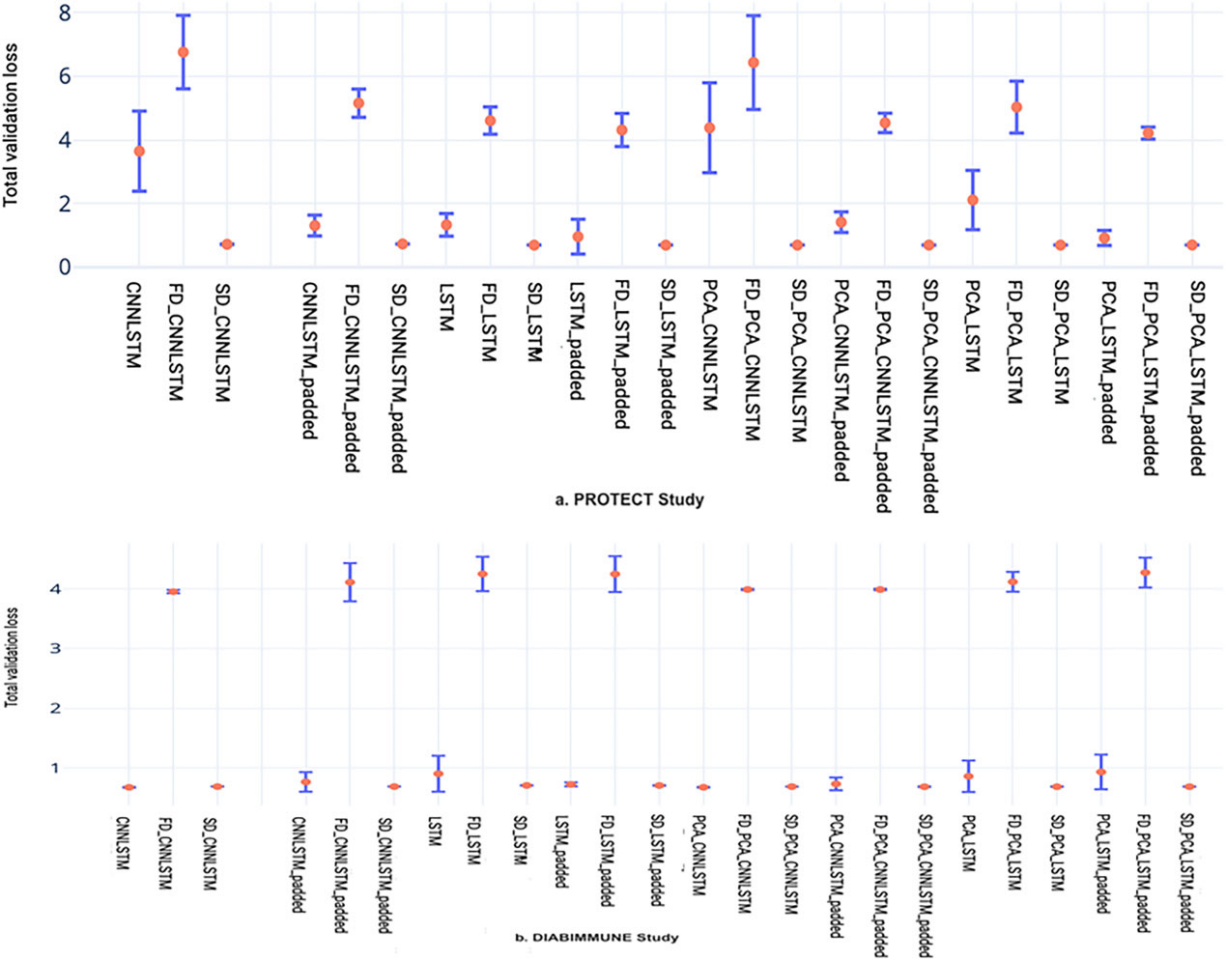
Results of different models of 10-fold cross-validation. (**a**) PROTECT study; (**b**) DIABIMMUNE study. SD, second distillation; FD, first distillation; Padded, padded in sequence; CNN, convolutional neural network; LSTM, long short-term memory; PCA, principal component analysis

The averaged validation loss of the models’ training on the PROTECT study and DIABIMMUNE study can be found in Figure 2. We can see that in both PROTECT study and DIABIMMUNE study, the validation loss for the first distillation (FD)-based loss and the PCA-based loss tends to be higher. This is due to the multiple sub-classifiers that try to learn the main classifier prediction in addition with the feature loss and the KL divergence. The validation loss for the second distillation loss is lower due to the fact that the model is getting a percentage of the total loss from its previous epoch prediction as a regularization where the model can be already good at performing the prediction.

### 3.2 Prediction performance based on the PROTECT study

In this analysis, we only obtained the OTU features and removed all other information about the patients. Each patient contains either stool samples or biopsy samples at week 0, week 4, week 12 and week 52. A large proportion of the patients have missing samples at different time points/weeks. We solved this by using either one of the three methods that we mentioned in Section 2.1.2. We used the subject’s disease severity as the disease outcomes for prediction using the networks based on the OTUs features. The disease severity includes three labels: *inactive*, *mild*, *moderate*, *severe*.

We split the dataset into 10-fold cross-validation to evaluate our models. We also evaluated our models on the different data pre-processing method and determined which one shows the best performance (Table 2). Table 2 shows the prediction performance of the 10-fold cross-validation on the PROTECT study. CNN-LSTM with padding in sequence achieves the best performance with AUC score 0.889 when compared with the other models.

**Table 2.** Prediction performance of the 10-fold cross-validation on the PROTECT study without self-distillation

Model	PCA	Preprocessing	Model performance			
			AUC mean	AUC stdev	F1 mean	F1 stdev
LSTM	TRUE	Pad in sequence	0.861	0.041	0.69	0.059
		Pad at end	0.792	0.041	0.59	0.042
	FALSE	Pad in sequence	0.864	0.054	0.685	0.064
		Pad at end	0.789	0.038	0.58	0.036
CNN-LSTM	TRUE	Pad in sequence	0.877	0.045	**0.717**	0.057
		Pad at end	0.814	0.024	0.612	0.025
	FALSE	Pad in sequence	**0.889**	0.042	0.716	0.057
		Pad at end	0.802	0.03	0.598	0.033

PCA, principal component analysis; stdev, standard deviation.

Bolded texts are the highest performance.

**Fig. 3.**
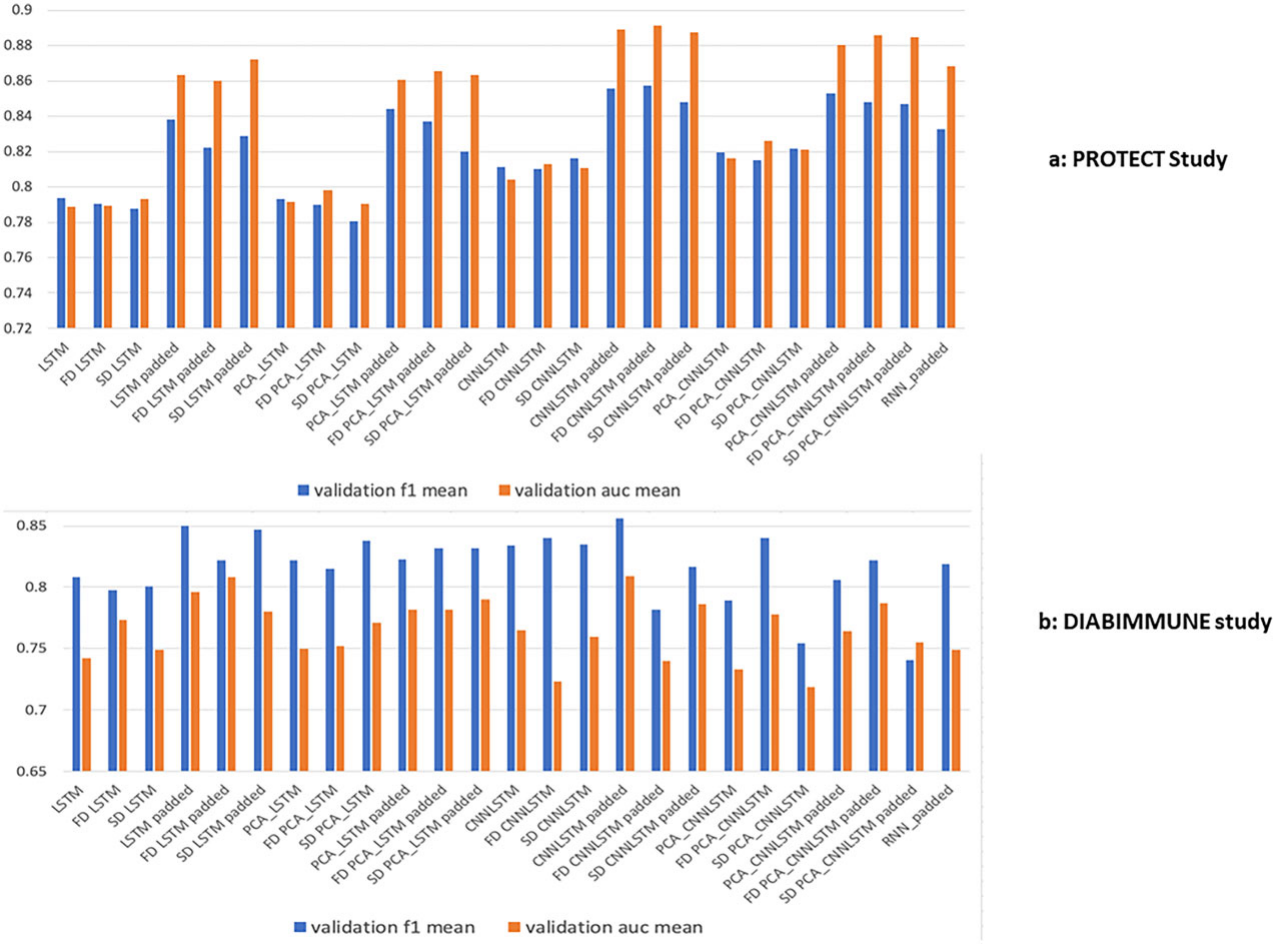
Prediction performance of the 10-fold cross-validation. (**a**) PROTECT study. Padded are ones that we evaluated on the prediction on week 52 (the last time points of each subject). For subjects that do not have values at week 52, they were removed. The models without padded were evaluated by obtaining the last available time points each subject has. (**b**) DIABIMMUNE study. Models with padded were evaluated on the max time points (max time points is the last time points available among all the subjects). The subjects that do not have the last available time points were removed. Models without padded were evaluated on the subject’s last available time points. SD, second distillation; FD, first distillation; Padded, padded in sequence; CNN, convolutional neural network; LSTM, long short-term memory; PCA, principal component analysis

We evaluated self-distillation on the PROTECT study. As shown in Figure 3a, we can see the self-distillation techniques improve the baseline performance. The first distillation improves most of the baseline model performance. The best performing model is the CNN-LSTM padded in sequence with first distillation achieving 0.89 AUC and 0.86 F1 score while the second distribution for the CNN-LSTM padded in sequence achieves the second best performance. The baseline models that do not use the self-distillation and the padding technologies show the worse performance.

### 3.3 Prediction based on the DIABIMMUNE study

In this analysis, we used 282 OTU features as input to the models. There are 203 subjects and 3 different allergies: *milk*, *egg* and *peanut.* Fifty-three of them have milk allergies, 40 of them have egg allergies, 9 of them have peanut allergies and the rest does not have any allergies. Each subject has a total of 38-time steps.

We split the dataset into 10-fold cross-validation and evaluated our models based on the split. The results can be seen in Table 3.

**Table 3.** Prediction performance of the 10-fold cross-validation on the DIABIMMUNE study without self-distillation

Model	PCA	Preprocessing	Model performance			
			AUC mean	AUC stdev	F1 mean	F1 stdev
LSTM	TRUE	Pad in sequence	0.783	0.068	0.456	0.109
		Pad at end	0.751	0.079	0.437	0.133
	FALSE	Pad in sequence	0.795	0.068	0.481	0.134
		Pad at end	0.742	0.073	0.428	0.117
CNN-LSTM	TRUE	Pad in sequence	0.754	0.084	0.443	0.17
		Pad at end	0.723	0.071	0.416	0.139
	FALSE	Pad in sequence	**0.798**	0.764	**0.508**	0.111
		Pad at end	0.764	0.764	0.464	0.116

Bolded texts are the highest performance.

In the DIABIMMUNE study, the best performing model is the CNN-LSTM model with no PCA, achieving 0.798 AUC score and 0.508 F1 score. We also evaluated self-distillation on the DIABIMMUNE study. As shown in Figure 3b, we can see that the first self-distillation method was able to improve most of the baseline methods. The result is consistent with that of the PROTECT study. The best performing model in the DIABIMMUNE study is the LSTM padded in sequence with first distillation and CNNLSTM padded in sequence achieving 0.81 AUC, 0.82 F1 score and 0.81 AUC, 0.86 F1 score, respectively.

## 4 Discussion

### 4.1 Comparison with other deep learning models

We also compared our best performing models with an existing deep learning network that uses LSTM to predict allergies longitudinal microbiome taxonomic profiles (Metwally *et al.*, 2019). To prevent from confusion, we will call this existing deep learning network as U-LSTM. We trained their model on the PROTECT and DIAIBIMMUNE datasets and compared the results against those from our models. We also created a RNN by replacing LSTM to RNN in our network to compare its performance with our models. As shown in Table 4, our model (CNNLSTM_padded + FD) outperforms the U-LSTM and RNN models, and achieves the highest AUC value of 0.89 and F1 score 0.86 in the PROTECT study.

**Table 4.** Performance comparison of our best model on 10-fold cross-validation in each dataset with other baseline models

PROTECT				
Model	AUC mean	AUC stdev	F1 mean	F1 stdev
**CNNLSTM_padded + FD**	**0.89**	0.04	**0.86**	0.037
U-LSTM	0.74	0.04	0.74	0.03
RNN	0.87	0.04	0.83	0.03

DIABIMMUNE				

Model	AUC mean	AUC stdev	F1 mean	F1 stdev

**LSTM_padded + FD**	**0.81**	0.07	**0.82**	0.06
U-LSTM	0.62	0.1	0.58	0.13
RNN	0.75	0.07	0.82	0.07

Bolded texts are the highest performance.

### 4.2 Sensitivity analysis

We ran sensitivity analysis to evaluate how much of the time in the past is relevant in predicting the outcome of the disease for both the DIABIMMUNE and PROTECT datasets. We used CNNLSTM_padded + FD on the PROTECT study and LSTM_padded + FD on the DIABIMMUNE study. The months in the DIABIMMUNE study are the number of past months used to predict the outcome of the disease. As shown in Table 5, we can see that the performance does not deteriorate that much when the number of past months is larger than 3. When the number of months is 3 or less, the performance is reduced by a visible amount. In the PROTECT study, the time points are (0, 4, 12, 52) weeks on which biopsy samples were only included in week 0 and week 52 while stool samples were included in all time points (6 time points in total). There is no visible difference in reduction of the performance with different time points used.

**Table 5.** Sensitivity analysis on the effect of time on our best models’ performance

PROTECT				
Time points	Auction mean	Auction stdev	F1 mean	F1 stdev
5	0.89	0.04	0.85	0.03
4	0.88	0.05	0.85	0.03
3	0.89	0.04	0.86	0.05

DIABIMMUNE				

Months	AUC mean	AUC stdev	F1 mean	F1 stdev

13	0.77	0.07	0.83	0.07
12	0.77	0.07	0.83	0.08
11	0.78	0.07	0.81	0.1
10	0.77	0.07	0.81	0.1
9	0.77	0.07	0.81	0.1
8	0.78	0.07	0.81	0.1
7	0.77	0.06	0.81	0.1
6	0.78	0.07	0.81	0.1
5	0.78	0.07	0.81	0.1
4	0.78	0.07	0.80	0.1
3	0.74	0.05	0.78	0.1
2	0.74	0.06	0.76	0.1

In addition, we ran transfer learning to determine if there is any performance improvement by transferring the features learned from the PROTECT study to the DIABIMMUNE study. As the PROTECT study achieves AUC higher than the DIABIMMUNE study. We decided to use the features learned from the PROTECT study to transfer to the DIABIMMUNE study to evaluate if performance can be further improved. The transfer learning did not show significant improvement on the DIABIMMUNE study when transferring the learned features from the PROTECT study (results are not shown).

## 5 Conclusion

Deep learning is able to classify disease severity and allergy reaction based on the longitudinal gut microbiome as shown in our experiments. Using temporal dynamics of the gut microbiome, we are able to capture more information about the longitudinal gut microbiome to gain a richer feature representation of the gut microbiome to classify the disease severity in the PROTECT study and the allergy reaction in the DIABIMMUNE study. Using CNN in combination with LSTM helps to improve the performance in achieving higher AUC score as evaluated in both the PROTECT study and the DIABIMMUNE study. Our experiments show that self-distillation is able to improve the performance of the LSTM in both the PROTECT study and the DIABIMMUNE study. Using data imputation does not necessarily improve the deep learning model’s performance. In the PROTECT study and the DIABIMMUNE study, the best performing models are the ones without any data imputation. We also compared our models against other existing deep learning model for longitudinal gut microbiome studies and RNN, and showed that our model has better performance than these deep learning-based models.

One limitation of our study is that we used class level annotations to extract the same features in both datasets for the transfer learning. Prior work has shown the class level to be too granular for machine learning classification. Even so, we still show this strategy has better performance than other baseline models, further demonstrating the elegancy of our modelling framework. In the future, we will further explore this issue using OTU level data.

In conclusion, we show that the CNN combined with the LSTM can achieve better generalizing models to classify disease severity and allergy reaction of subjects. With the addition of self-distillation, it is able to achieve the highest performing models in both the PROTECT study and the DIABIMMUNE study for the LSTM model. As for the CNNLSTM model, it was able to improve the performance in the PROTECT study, the performance for the DIABIMMUNE study remains no significant changes when self-distillation was incorporated.

## References

[vbad059-B1] Andrews S. et al (2003) Support vector machines for multiple-instance learning. Adv. Neural Inf. Process. Syst., **15**.

[vbad059-B2] Bogart E. et al (2019) MITRE: inferring features from microbiota time-series data linked to host ltatus. Genome Biol., 20, doi:10.1186/s13059-019-1788-y PMC6721208 31477162

[vbad059-B3] Bokulich N.A. et al (2016) Antibiotics, birth mode, and diet shape microbiome maturation during early life. Sci. Transl. Med., 8, doi:10.1126/scitranslmed.aad7121 PMC5308924 27306664

[vbad059-B4] Breiman L. (2001) Random forests. Mach. Learn., 45, 5–32, doi:10.1023/A:1010933404324

[vbad059-B5] Brooks B. et al (2017) Strain-resolved analysis of hospital rooms and infants reveals overlap between the human and room microbiome. Nat. Commun., 8, doi:10.1038/s41467-017-02018-w PMC5703836 29180750

[vbad059-B6] Bunescu R.C. , Mooney R.J. (2007) Multiple instance learning for sparse positive bags. In: *ACM International Conference Proceeding Series*. doi:10.1145/1273496.1273510

[vbad059-B7] Chen X. et al (2021) Human host status inference from temporal microbiome changes via recurrent neural networks. Brief. Bioinf., 22, doi:10.1093/bib/bbab223 34151933

[vbad059-B8] Cho K. et al (2014) Learning phrase representations using RNN encoder-decoder for statistical machine translation. In: *Proceedings of the EMNLP 2014—2014 Conference on Empirical Methods in Natural Language Processing.* doi:10.3115/v1/d14-1179

[vbad059-B9] Cortes C. , Vapnik V. (1995) Support-vector networks. Mach. Learn., 20, 273–297, doi:10.1023/A:1022627411411

[vbad059-B10] David L.A. et al (2014) Diet rapidly and reproducibly alters the human gut microbiome. Nature, 505, 559–563, doi:10.1038/nature12820 24336217 PMC3957428

[vbad059-B11] DiGiulio D.B. et al (2015) Temporal and spatial variation of the human microbiota during pregnancy. Proc. Natl. Acad. Sci. USA, 112, 11060–11065, doi:10.1073/pnas.1502875112 26283357 PMC4568272

[vbad059-B12] García-Jiménez B. et al (2021) Predicting microbiomes through a deep latent space. Bioinformatics (Oxford, England), 37, 1444–1451, doi:10.1093/bioinformatics/btaa971 33289510 PMC8208755

[vbad059-B13] Gilbert J.A. et al (2018) Current understanding of the human microbiome. Nat. Med., 24, 392–400, doi:10.1038/nm.4517 29634682 PMC7043356

[vbad059-B14] Hall A.B. et al (2017) A novel Ruminococcus gnavus clade enriched in inflammatory bowel disease patients. Genome Med., 9, doi:10.1186/s13073-017-0490-5 PMC5704459 29183332

[vbad059-B15] Handelsman J. (2004) Metagenomics: application of genomics to uncultured microorganisms. Microbiol. Mol. Biol. Rev., 68, 669–685, doi:10.1128/MMBR.68.4.669-685.2004 15590779 PMC539003

[vbad059-B16] Heintz-Buschart A. et al (2016) Integrated multi-omics of the human gut microbiome in a case study of familial type 1 diabetes. Nat. Microbiol., 2, doi:10.1038/nmicrobiol.2016.180 27723761

[vbad059-B17] Ho T.K. (1995) Random decision forests. In: *Proceedings of the International Conference on Document Analysis and Recognition*, ICDAR. doi:10.1109/ICDAR.1995.598994

[vbad059-B18] Hochreiter S. , Schmidhuber J. (1997) Long short-term memory. Neural Comput., 9, 1735–1780. 9377276 10.1162/neco.1997.9.8.1735

[vbad059-B19] Hyams J.S. et al (2017) Factors associated with early outcomes following standardised therapy in children with ulcerative colitis (PROTECT): a multicentre inception cohort study. Lancet Gastroenterol. Hepatol., 2, 855–868, doi:10.1016/S2468-1253(17) 28939374 PMC5695708

[vbad059-B151] Kim K. et al (2020) Self-Knowledge distillation with progressive refinement of targets. In: 2021 IEEE/CVF International Conference on Computer Vision (ICCV), pp. 6547–6556.

[vbad059-B21] Kotzias D. et al (2015) From group to individual labels using deep features. In: *Proceedings of the ACM SIGKDD International Conference on Knowledge Discovery and Data Mining*. doi:10.1145/2783258.2783380

[vbad059-B22] Krizhevsky A. et al (2012) ImageNet classification with deep convolutional neural networks. In Pereira, F. *et al.* (ed.) *Advances in Neural Information Processing Systems 25*. Curran Associates, Inc., pp. 1097–1105.

[vbad059-B23] Kullback S. , Leibler R.A. (1951) On information and sufficiency. Ann. Math. Statist., 22, 79–86, doi:10.1214/aoms/1177729694

[vbad059-B24] LaPierre N. et al (2019) MetaPheno: a critical evaluation of deep learning and machine learning in metagenome-based disease prediction. Methods, 166, 74–82, doi:10.1016/j.ymeth.2019.03.003 30885720 PMC6708502

[vbad059-B25] Maarastawi S.A. et al (2018) Crop rotation and straw application impact microbial communities in Italian and Philippine Soils and the rhizosphere of *Zea mays*. Front. Microbiol., 9, 1295, doi:10.3389/fmicb.2018.01295 29963033 PMC6013709

[vbad059-B26] McIver L.J. et al (2018) bioBakery: a meta’omic analysis environment. Bioinformatics, 34, 1235–1237, doi:10.1093/bioinformatics/btx754 29194469 PMC6030947

[vbad059-B27] Metwally A.A. et al (2019) Utilizing longitudinal microbiome taxonomic profiles to predict food allergy via long short-term memory networks. PLoS Comput. Biol., 15, e1006693, doi:10.1371/journal.pcbi.1006693 30716085 PMC6361419

[vbad059-B28] Ng A. (2011) Sparse autoencoder. CS294A Lecture Notes, 72, 1–19.

[vbad059-B29] Pasolli E. et al (2016) Machine learning meta-analysis of large metagenomic datasets: tools and biological insights. PLoS Comput. Biol., 12, e1004977, doi:10.1371/journal.pcbi.1004977 27400279 PMC4939962

[vbad059-B30] Pasolli E. et al (2017) Accessible, curated metagenomic data through ExperimentHub. Nat. Methods, 14, 1023–1024, doi:10.1038/nmeth.4468 29088129 PMC5862039

[vbad059-B31] Pearson K. (1901) LIII. On lines and planes of closest fit to systems of points in space. Lond. Edinburgh Dublin Philos. Mag. J. Sci., 2, 559–572, doi:10.1080/14786440109462720

[vbad059-B32] Qin J. et al; MetaHIT Consortium. (2010) A human gut microbial gene catalogue established by metagenomic sequencing. Nature, 464, 59–65, doi:10.1038/nature08821 20203603 PMC3779803

[vbad059-B33] Qin N. et al (2014) Alterations of the human gut microbiome in liver cirrhosis. Nature, 513, 59–64, doi:10.1038/nature13568 25079328

[vbad059-B34] Rabiner L.R. , Juang B.H. (1986) An introduction to hidden Markov models. IEEE ASSP Mag., 3, 4–16, doi:10.1109/MASSP.1986.1165342

[vbad059-B35] Rahman M.A. , Rangwala H. (2018) RegMIL: phenotype classification from metagenomic data. In: *ACM-BCB 2018—Proceedings of the 2018 ACM International Conference on Bioinformatics, Computational Biology, and Health Informatics*. doi:10.1145/3233547.3233585

[vbad059-B36] Raymond F. et al (2016) The initial state of the human gut microbiome determines its reshaping by antibiotics. ISME J., 10, 707–720, doi:10.1038/ismej.2015.148 26359913 PMC4817689

[vbad059-B37] Rumelhart D.E. et al (2013) Learning internal representations by error propagation. In: *Readings in Cognitive Science: A Perspective from Psychology and Artificial Intelligence*. doi:10.1016/B978-1-4832-1446-7.50035-2

[vbad059-B38] Schirmer M. et al (2018) Compositional and temporal changes in the gut microbiome of pediatric ulcerative colitis patients are linked to disease course. Cell Host Microbe, 24, 600–610.e4, doi:10.1016/j.chom.2018.09.009 30308161 PMC6277984

[vbad059-B39] Shao Y. et al (2019) Stunted microbiota and opportunistic pathogen colonization in caesarean-section birth. Nature, 574, 117–121, doi:10.1038/s41586-019-1560-1 31534227 PMC6894937

[vbad059-B40] Sharma D. , Xu W. (2021) phyLoSTM: a novel deep learning model on disease prediction from longitudinal microbiome data. Bioinformatics, 37, 3707–3714, doi:10.1093/bioinformatics/btab482 34213529

[vbad059-B41] Simonyan K. , Zisserman A. (2015) Very deep convolutional networks for large-scale image recognition. CoRR, abs/1409.1556.

[vbad059-B42] Tadeusiewicz R. (1995) Neural networks: a comprehensive foundation. Control Eng. Pract., 3, 746–747, doi:10.1016/0967-0661(95)90080-2

[vbad059-B43] Tibshirani R. (1996) Regression shrinkage and selection via the lasso. J. R. Stat. Soc. Ser. B (Methodological), 58, 267–288, doi:10.1111/j.2517-6161.1996.tb02080.x

[vbad059-B44] Vatanen T. et al (2016) Variation in microbiome LPS immunogenicity contributes to autoimmunity in ‘humans’. Cell, 165, 1551, doi:10.1016/j.cell.2016.04.007 27259157

[vbad059-B45] Vincent C. et al (2016) Bloom and bust: intestinal microbiota dynamics in response to hospital exposures and *Clostridium difficile* colonization or infection. Microbiome, 4, doi:10.1186/s40168-016-0156-3 PMC4791782 26975510

[vbad059-B46] Walters W.A. et al (2018) Large-scale replicated field study of maize rhizosphere identifies heritable microbes. Proc. Natl. Acad. Sci. USA, 115, 7368–7373, doi:10.1073/pnas.1800918115 29941552 PMC6048482

[vbad059-B47] Wang J. et al (2012) A metagenome-wide association study of gut microbiota in type 2 diabetes. Nature, 490, 55–60, doi:10.1038/nature11450 23023125

[vbad059-B48] Xie S. et al (2017) Aggregated residual transformations for deep neural networks. In: *Proceedings—30th IEEE Conference on Computer Vision and Pattern Recognition, CVPR 2017*. doi:10.1109/CVPR.2017.634

[vbad059-B49] Zhang L. et al (2019) Be your own teacher: improve the performance of convolutional neural networks via self distillation. In: *Proceedings of the IEEE International Conference on Computer Vision*. doi:10.1109/ICCV.2019.00381

